# Tetanus, diphtheria and pertussis vaccination and risk for incident dementia among adults with down syndrome

**DOI:** 10.1016/j.tjpad.2026.100583

**Published:** 2026-05-05

**Authors:** Kimberly Schiel, Joanne Salas, Anjani Urban, Daniel F. Hoft, Jeffrey F. Scherrer

**Affiliations:** a Department of Family and Community Medicine, Saint Louis University School of Medicine, 1008 S. Spring, St. Louis, MO 63110 USA; b Advanced HEAlth Data (AHEAD) Research Institute, Saint Louis University School of Medicine, 3545 Lafayette Ave, 4th Floor, St. Louis, MO 63104 USA; c Department of Health and Clinical Outcomes Research, Saint Louis University School of Medicine. Salus Center, 4th Floor, 3545 Lafayette Ave. St. Louis, MO 63104 USA; d Department of Internal Medicine Division of Infectious Diseases, Allergy, and Immunology, Saint Louis University School of Medicine, Saint Louis, MO. United States; e Department of Molecular Microbiology & Immunology, Saint Louis University School of Medicine, Saint Louis, MO. United States; f Department of Psychiatry and Behavioral Neuroscience, Saint Louis University School of Medicine, 1438 South Grand Blvd., St. Louis, MO 63104 USA

**Keywords:** Down syndrome, Vaccination, Alzheimer’s disease, Dementia, Epidemiology

## Abstract

**Background:**

Adult vaccination is inversely associated with incident Alzheimer’s Disease and Related Dementias.

**Objectives:**

We determined if Tetanus, Diphtheria and Pertussis (Tdap) vaccination was linked to incident Alzheimer’s Disease and dementia among adults with Down Syndrome, 50% of whom develop Alzheimer’s Disease by age 60.

**Design:**

This is a retrospective cohort study using TriNetX nationally distributed electronic health records from 2013 to 2024.

**Setting:**

Historical medical record data.

**Participants:**

5591 patients with Down Syndrome across the United States. Eligible patients were free of Alzheimer’s Disease and dementia prior to index. Index date could occur 1/1/2015 to 1/1/2020 allowing for 5 to 10 years of possible follow-up time.

**Measurements:**

Vaccination was measured using product name and procedure codes and Alzheimer’s Disease and dementias was defined by ICD-10 codes.

**Results:**

The mean age of the cohort was 50.0 (±8.3), 50.1% were female and 72.1% were White. After controlling for confounding, Tdap vaccination vs. remaining without Tdap vaccination was associated with lower Alzheimer’s Disease and dementia risk (HR=0.74; 95%CI:0.57–0.98).

**Conclusions:**

In a cohort of patients with Down Syndrome, Tdap vaccination was associated with a 26% lower risk for Alzheimer’s Disease and dementia. This is a novel and important finding because existing studies of vaccination and reduced risk for Alzheimer’s Disease and dementia have been among cognitively intact adults. This study reveals benefits of vaccination even among those at high risk for Alzheimer’s Disease and dementia due to Down Syndrome. Future studies are needed to understand the mechanisms underlying this relationship.

## 1 Background

Systematic reviews and meta-analyses indicate that receiving routine adult vaccinations is associated with reduced risk for Alzheimer’s Disease and Related Dementias (ADRD) [[1], [2], [3], [4]]. The reduction appears to be associated with all types of vaccines [1], and there may be a cumulative effect with receiving various types of vaccines [5]. Individual studies show that reduction in dementia risk is associated with herpes zoster vaccination [3,6], influenza vaccination [2,4] or Tdap vaccination [7]. The magnitude of reduced risk is substantial and much larger than any existing prevention and intervention modalities. Prior studies among older adults in the general population, without dementia at baseline, indicate that persons who receive Tetanus, Diphtheria and Pertussis (Tdap) vaccination, compared to those who remain without Tdap, have a 42% lower risk for incident dementia during a follow-up period of 6–7 years [7].

To our knowledge, vaccination and risk for ADRD has not been investigated in one population at extreme risk for dementia, which are people with Down Syndrome [8]. People with Down Syndrome have much higher rates of dementia than the general population, and they experience earlier onset of dementia [8,9]. Approximately 50% of people with Down Syndrome have dementia by the age of 60 [8], while the rate in the general population is <2% for those age 65–69 [10]. The mechanism is thought to be due to the triplication of Chromosome 21, on which is located the gene for amyloid protein precursor. Almost all people with Down Syndrome have higher levels of amyloid and evidence of amyloid plaques and neurofibrillary tangles occurring by age 40. The incidence of dementia in Down Syndrome is rare before age 40 but rises sharply afterward [8]. Given that the average life expectancy of a person with Down Syndrome is now approximately 60 year [11], it is likely that most people with Down Syndrome will experience dementia by the end of their lifespan. Dementia is a major contributor to the limited lifespan of people with Down Syndrome [12]. While there are clinical trials underway for strategies to prevent dementia in Down Syndrome [13,14], there are no current effective preventive strategies. Similarly, standard treatment for dementia has shown little to no efficacy in this population [15,16].

People with Down Syndrome display markedly dysregulated immune systems, with a relative immunodeficiency during childhood [17,18] and also later in life if they have neurologic disease such as seizures or dementia [19]. A link between immune dysregulation and early onset Alzheimer’s Disease is suspected, although the mechanism is not clear. Patients with Down Syndrome share many of the same cerebral spinal fluid protein alterations as those with late-onset AD, but changes involving immune-related proteins are more severe in patients with Down Syndrome. These changes may be a risk factor for development of ADRD [20].

Based on existing studies in adult populations, a promising approach to reducing risk for ADRD in Down Syndrome may be routine vaccination. Two recent natural experiments have reported the most robust evidence to date that vaccination is followed by and has a causal association with reduced ADRD risk. These natural experiments compared ADRD risk between those born just before vs. just after eligibility for herpes zoster vaccination in Wales [21] and Australia [22] and found those eligible for the vaccination has lower ADRD risk. However, this work did not consider Down Syndrome and was limited to older adults.

Whether adult vaccination reduces risk for ADRD among people with Down Syndrome is unknown. Given the large burden of dementia in people with Down Syndrome, and the potential protective effect of immunizations against dementia in the general population, we sought to determine whether adult Tdap immunization in people with Down Syndrome has the same protective effect. Using a nationally distributed electronic health record data base, we hypothesize that during our observation period, people with Down Syndrome who receive Tdap vaccination will have a significantly lower risk for ADRD compared to those who had no medical record documented Tdap vaccination.

## 2 Methods

Eligible patients were identified from the TriNetX Research Network (TriNetX LLC, Cambridge, MA, USA). Historical, de-identified data were downloaded on 3/3/2025 and included ICD-9 and ICD-10 diagnostic codes, Current Procedural Terminology (CPT) codes, prescription medication orders, vital signs, laboratory results, and demographic measures. Downloaded data included 74,827 patients from 87 healthcare organizations across the United States who had a Down Syndrome diagnosis and at least one ambulatory healthcare encounter from 2013 to 2024.

Because data were historical, did not involve interaction or intervention with human subjects, and de-identified per the de-identification standard in Section §164.514(a) of the HIPAA Privacy Rule, this study was deemed non-human subjects research and exempt from institutional review board approval.

**Fig. 1:**
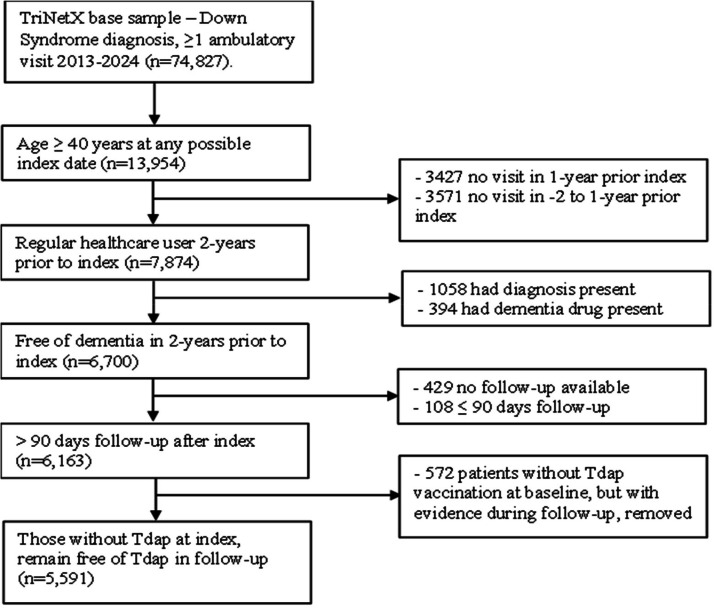
Sampling Scheme (rolling or dynamic cohort entry 1/1/15, 1/1/16….,1/1/20).

The downloaded data were further restricted based on analytic cohort specifications. The index date was the first date where a patient was:1) at least 40 years old; 2) without ADRD diagnoses; and 3) were regular healthcare users, (i.e. minimum of an annual visit) in the 2-years prior to index. The age criteria was applied to ensure patients were at risk for ADRD in follow-up. The yearly outpatient visit in the 2 year look back period was applied to increase likelihood that these were repeated users of the same health care system. We used a dynamic cohort design with 6 possible index dates from 1/1/2015 to 1/1/2020 (e.g. 1/1/15, 1/1/16….1/1/20), and the first date eligibility criteria were met was defined as index, i.e. baseline. Thus, all patients had a possible maximum of 60 to 120 months of follow-up time and all had a 2 year look-back period to measure potential confounders and to remove prevalent ADRD. Patients were excluded if they had ≤90 days follow-up to reduce the risk of subclinical ADRD transitioning to ADRD shortly after index. Additionally, following prior investigations of vaccination and incident ADRD [[5], [6], [7]] we conducted analyses as a per-protocol approach. Therefore, we removed 572 patients (out of 5635 non-Tdap patients =10.1%) who had not received a vaccination by index date but received one during follow-up.. After applying eligiblity criteria, there were 5591 patients with Down Syndrome and without an ADRD diagnosis at index date. See Fig. 1 for sampling approach

### 2.1 Study variables

Detailed variable definitions are shown in Appendix A, e-table 1. Down Syndrome was defined by ICD-10 codes starting with Q90 (Q90*).

**Exposure:** Tdap vaccination was measured using existing definitions we have employed in other studies of vaccination and dementia [5]. Specifically, Tdap vaccination was defined by CPT codes 90,701 or 90,715 and by product names Adacel and Boostrix. Tdap vaccination could occur anytime prior to index date. The non-Tdap group had to remain without medical record evidence of Tdap vaccination during follow-up. Two systematic reviews and meta-analyses [1,23] of vaccination and dementia risk indicates numerous vaccine types are associated with reduction in ADRD risk including herpes zoster, rabies, hepatitis A and B, influenza, pneumonia, typhoid and Tdap. Because there is no evidence that only one or two specific types of vaccinations are associated with ADRD risk, we focused on Tdap vaccinations. In addition, Tdap vaccination is much more likely to be accurately documented in the medical record and one that all patients would be eligible for during the observation time. Thus, we focused on a vaccine that is common in middle age and would be observable during our study period based on guideline Tdap vaccination schedule. In addition, herpes zoster and RSV is likely to be less prevalent given it is mostly administered to older adults. We did not model influenza because our experience suggests the frequent receipt of this vaccine in pharmacies, employers and health fairs leads to misclassifying the exposure.

**Outcome:** Incident dementia in follow-up was defined by ICD-9 and or ICD-10 diagnostic codes on two separate days in any 12-month period. The first of the two codes was considered date of onset. This definition has good agreement with Mini Mental State Exam and the Saint Louis University Mental Status Examination scores indicating mild or worse dementia [24,25]. We did not limit the outcome to Alzheimer's Disease given we do not know why physicians came to make a specific ADRD diagnosis (e.g., distinguishing unspecified dementia from Alzheimer’s Disease). Follow-up time was measured as months from index to incident ADRD or censor date, which for those without incident ADRD was the last available encounter date in follow-up.

**Potential confounders:** Confounders were selected based on existing studies identifying risk for dementia or vaccination [26,27]. All potential confounders were measured in the 2 years prior to index date. Demographics included age, sex, race and geographic region. We controlled for high health care utilization to reduce risk of detection bias. High utilization, based on number of visits, was defined as the top 25th percentile of health care users vs. not.

Physical comorbidities included type 2 diabetes, obesity, hypertension, stroke, ischemic heart disease, congestive heart failure, atrial fibrillation, congenital heart defect, hypothyroidism, traumatic brain injury, and vitamin B12 deficiency. Psychiatric comorbidities we controlled for included depression, a composite measure of anxiety disorders (any of the following: panic, obessessive compulsive disorder, social phobia, GAD, Anxiety NOS and posttraumatic stress disorder), and severe mental illness defined as bipolar disorder or schizophrenia.

We controlled for sustained use, defined as 2 or more prescriptions in any 6 month period [5], of NSAIDs, antihypertensives, statins, steroids, antivirals, metformin, sulfonylureas, antidepressants, atypical antipsychotics and benzodiazepines.

### 2.2 Analytic approach

Entropy balancing (E-balance) was applied to control for confounding and to balance covariates among those with and without Tdap vaccination [28]. E-balance removes differences in the distribution of potential confounders by vaccination status, similar to the use of propensity scores (PS) and inverse probility of exposure weighting (IPEW). E-balance, compared to these other common methods like PS and IPEW, can achieve superior covariate balance without being dependent on model specification by deriving weights to ensure that specified covariate moments (i.e., mean and variance) are approximately equal across groups. Standardized mean difference (SMD) percent (SMD%=100*SMD) was used to evaluate balance before and after weighting, with an SMD% < 10% indicating succesful balance [29]. The *WeightIt* package in R-Studio (version 4.2.2) was used to compute e-balance weights for the average treatment effect.

All remaining, primary analyses were conducted using SAS version 9.4 (SAS Institute, Cary, NC). Descriptive statistics were presented as means and standard deviations or frequencies and percents. Chi-square tests and independent samples *t*-tests assessed bivariate associations of covariates with Tdap vaccination status at baseline, and covariate balance before and after e-balance weighting was assessed with SMD%. Chi-square tests and Poisson models with an offset for log(follow-up time) compared crude cumulative incidence and incidence rate of ADRD, respectively, between Tdap vaccination status groups. Cox proportional hazards models, before and after e-balance weighting to control for confounding, calculated hazard ratios (HR) and 95% confidence intervals (CI) for the relationship of Tdap vaccindation and time to incident ADRD. Weighted models incorporated robust, sandwich-type variance estimators to calculate CI’s and p-values [29]. Proportional hazard assumptions were met for all models (p>.05).

To determine if unmeasured confounding may explain results, we computed the e-value [30]. The e-value is the magnitude of association between an unmeasured confounder and both the exposure and outcome which would account for the association between Tdap and ADRD. For example, if the e-value for a HR is 1.5, the relationship of a potential unmeasured confounder must be at least an HR=1.5 for both the exposure and outcome for the exposure and outcome relationship to be null (i.e. HR=1 OR = 1.5 or RR=1.5). Further test of robustness was done using a negative control analysis by computing the relationship between vaccination and a new diagnosis for gall stones. Last, we conducted an intention to treat analyses and dropped the 90-day follow-up criteria. Patients with Tdap in follow-up were removed if they were without Tdap at baseline (n = 6271).

## 3 Results

As reported in Table 1, receipt of a Tdap vaccination was documented for 9.5% of the sample. Overall, the mean age of the cohort was 50.0 (±8.3), 50.1% were female, 72.1% were White and 10.1% Black race. The most prevalent comorbidities were obesity (33.3%), hypothyroidism (29.1%), and anxiety disorders (9.6%).

**Table 1:** Demographic and baseline characteristics overall and by Tdap vaccination status at index (n = 5591).

Covariates, n(%)	Overall (n = 5591)	No Tdap (n = 5063)	Tdap (n = 528)	p-value	SMD%
Age, mean (±sd)	50.0 (±8.3)	50.2 (±8.3)	47.3 (±8.3)	<0.0001	35.7
Sex					
Female	2802 (50.1)	2506 (49.5)	296 (56.1)	.0003	13.2
Male	2477 (44.3)	2257 (44.6)	220 (41.7)		5.9
Unknown	312 (5.6)	300 (5.9)	12 (2.3)		18.5
Race					
White	4032 (72.1)	3651 (72.1)	381 (72.2)	.423	0.1
Black	564 (10.1)	505 (10.0)	59 (11.2)		3.9
Other	419 (7.5)	376 (7.4)	43 (8.1)		2.7
Unknown	576 (10.3)	531 (10.5)	45 (8.5)		6.7
Region					
Midwest	1070 (19.1)	930 (18.4)	140 (26.5)	<0.0001	19.6
Northeast	2218 (39.7)	2073 (40.9)	145 (27.5)		28.7
South	1394 (24.9)	1264 (25.0)	130 (24.6)		0.8
West	654 (11.7)	576 (11.4)	78 (14.8)		10.1
Unknown	255 (4.6)	220 (4.4)	35 (6.6)		10.0
High healthcare utilization	1414 (25.3)	1153 (22.8)	261 (49.4)	<0.0001	57.8
Type II Diabetes	557 (10.0)	496 (9.8)	61 (11.6)	.199	5.7
Obesity	1861 (33.3)	1617 (31.9)	244 (46.2)	<0.0001	29.6
Hypertension	695 (12.4)	601 (11.9)	94 (17.8)	<0.0001	16.8
Stroke	87 (1.6)	81 (1.6)	6 (1.1)	.577	4.0
Ischemic heart disease	151 (2.7)	128 (2.5)	23 (4.4)	.014	10.0
Congestive heart failure	227 (4.1)	199 (3.9)	28 (5.3)	.128	6.5
Atrial fibrillation	102 (1.8)	91 (1.8)	11 (2.1)	.640	2.1
Congenital heart defect	467 (8.4)	414 (8.2)	53 (10.0)	.141	6.5
Hypothyroidism	1624 (29.1)	1435 (28.3)	189 (35.8)	.0003	16.0
Traumatic brain injury	79 (1.4)	66 (1.3)	13 (2.5)	.048	8.5
Vitamin B12 deficiency	128 (2.3)	101 (2.0)	27 (5.1)	<0.0001	16.9
Depression	408 (7.3)	322 (6.4)	86 (16.3)	<0.0001	31.7
Anxiety disorder^a^	536 (9.6)	434 (8.6)	102 (19.3)	<0.0001	31.4
Severe mental illness^b^	123 (2.2)	107 (2.1)	16 (3.0)	.162	5.8
NSAIDs^c^	815 (14.6)	688 (13.6)	127 (24.1)	<0.0001	27.0
Antihypertensives^c^	822 (14.7)	730 (14.4)	92 (17.4)	.063	8.2
Statins^c^	536 (9.6)	482 (9.5)	54 (10.2)	.599	2.4
Steroids^c^	1024 (18.3)	905 (17.9)	119 (22.5)	.008	11.6
Antivirals^c^	176 (3.2)	142 (2.8)	34 (6.4)	<0.0001	17.4
Metformin^c^	178 (3.2)	157 (3.1)	21 (4.0)	.275	4.7
Sulfonylurea^c^	67 (1.2)	57 (1.1)	10 (1.9)	.137	6.3
Antidepressant medications^c^	654 (11.7)	559 (11.0)	95 (18.0)	<0.0001	19.8
Atypical antipsychotics^c^	284 (5.1)	255 (5.0)	29 (5.5)	.650	2.0
Benzodiazepines^c^	489 (8.8)	441 (8.7)	48 (9.1)	.768	1.3

Note: SMD%=standardized mean difference percent (SMD*100).

a Anxiety disorders = panic disorder, OCD, social phobia, GAD, Anxiety NOS, PTSD.

b Severe mental illness = bipolar disorder or schizophrenia.

c Medications = sustained use prior to index (at least 2 prescription orders in any 6-month period).

Prior to control for confounding using e-balance, the mean age was significantly lower in the vaccinated group (SMD%=35.7) and female sex was more common in those with a Tdap vaccination (SMD%=13.2). High healthcare utilizers were more prevalent in vaccinated patients (SDM%=57.8). Obesity, hypertension, hypothyroidism, vitamin B12 deficiency, depression, and anxiety disorder were all more prevalent in the Tdap recipients (SMD% range:11.6 to 57.8).

E-balance effectively removed differences in the distribution of potential confounders by vaccination status, see Appendix, e-table 2. As shown in Table 2, the overall median follow-up time from index to incident dementia or end of follow-up was 67 months (IQR:41–94). Among those without Tdap, median follow-up time was 66 months (IQR 39–93) and among Tdap vaccinated it was 74.5 months (IQR: 56–108). Tdap vaccination was significantly associated with lower dementia cumulative incidence and incidence rate. The cumulative incidence of dementia among the overall sample was 13.9%, among non-Tdap vaccinated patients it was 14.4% and among those with a Tdap vaccination it was 9.8%.

**Table 2:** Dementia events - cumulative incidence % and incidence rate per 10,000 person-years (PY) (n = 5591).

Group	Observation time, median (IQR) – months a	Total n	Dementia events	Cumulative incidence %	Incidence rate per 10,000PY
*Overall*	*67 (41–94)*	*5591*	*779*	*13.9%*	*251.0/10,000PY*
No Tdap	66 (39–93)	5063	727	14.4%	262.8/10,000PY
Tdap	74.5 (56–108)	528	52	9.8%	154.0/10,000PY
				*p=.004*	*p=.002*

Note: PY=person-years; IQR = interquartile range.

a Observation time = Time to dementia or censoring.

Prior to controlling for confounding, patients who received a Tdap vaccination had a 42% lower risk for incident dementia (Table 3). After controlling for confounding using e-balance, this association remained significant (HR=0.74; 95%CI:0.57–0.98).

**Table 3:** Results from cox proportional hazard models estimating the association of Tdap vaccination on incident dementia (n = 5591).

	Model 1 – Crude/unweighted	Model 2 – Weighted ^a^
Group	Crude HR (95%CI)	Weighted HR (95%CI)
No Tdap	1.00	1.00
Tdap	0.58 (0.44–0.77)	0.74 (0.57–0.98)
	*p=.0001*	*p=.020*

Note: HR=hazard ratio; CI=confidence interval.

There was no association between Tdap vaccination and new gallstone diagnoses in negative outcome control analyses (HR=1.01; 95%CI:0.62–1.66). In other sensitivity analyses, we removed the exclusion criteria of ≤90 days follow-up and found that after dropping the ≤90 days follow-up criteria, those without Tdap had an ADRD incidence rate of 257.5/10,000PY as compared to 157.0/10,000PY among patients who received Tdap. After controlling for confounding using e-balance those who received Tdap were less likely to develop ADRD (HR = 0.79 (0.61–1.01).

## 4 Discussion

In a nationally distributed cohort of adults diagnosed with Down Syndrome, those who received Tdap vaccination had a 26% lower risk for incident ADRD compared to those who remained without Tdap over a 5 to 10 year follow-up period. To our knowledge, this is the first study to demonstrate vaccination in Down Syndrome is linked to a reduced risk for ADRD. This is an important finding because prior studies of vaccination and dementia in the general adult population have not measured Down Syndrome. The general adult population has a relatively small risk for incident dementia, while the rate of incident dementia in people with Down Syndrome is very high after age 40. With such high pre-existing risk, observing a benefit from an inexpensive, easily accessible Tdap vaccination highlights the potential for a new approach toward ADRD reduction in populations with or without Down Syndrome.

The biological mechanism for Tdap protection against incident dementia is unknown. It has been hypothesized to be related to a reduction in sytemic inflammation, which is known to be a risk factor for progression of neurodegenerative diseases [31]. Support for this hypothesis is provided by a study which shows that severe infections that do not involve the CNS but cause systemic inflammation increased risks of dementia [32]. Tdap may protect against inflammatory episodes in the patient’s life, either by preventing infections, or via improved control of inflammation induced by general immune training promoted by vaccination. People with Down Syndrome have challenges with immune dysregulation and increased susceptibility to infection[33], and so would benefit at least as much as their peers from avoiding vaccine-preventable illnesses.

It is possible that Tdap alone does not reduce risk for dementia but rather it is a proxy for receipt of other appropriate vaccinations that train the immune system. However this is speculative because we lack a lifetime vaccination history given the limited duration of our observation time. Yet it is logical that persons who receive Tdap are also more likely to have other appropriate vaccinations throughout life. This could reduce inflammation and improve patients’ ability to resist infection. Another explanation for benefits of vaccination on ADRD risk in adults without Down Syndrome is healthy patient bias.

One potential challenge in implementing these results may be possible vaccine hesitancy among families and caretakers of persons with Down Syndrome. The rates of adult vaccination among people with Down Syndrome is unknown, but there is evidence of vaccine hesitancy in the childhood years. There is a lower than average rate of general childhood immunizations for children with Down Syndrome [34], and a lower rate of childhood immunization against influenza [35]. In addition, US adults with disabilities have lower rates of seasonal influenza vaccine than adults without disabilities [36]. If vaccine hesitancy among people with Down Syndrome persists into the adulthood years, then they may not comply with adult vaccine recommendations, thereby missing the potential to lower dementia risk.

We acknowledge that, with a median follow-up time of 67 months, many patients in the study may have gone on to develop ADRD at a later time. It is not known whether Tdap vaccination would reduce death from ADRD or increase lifespan.

## 5 Limitations

There were risks of misclassification for the patient’s dementia and Tdap status. Misclassification of the patient’s type of dementia could bias results. If we missed cases of Tdap in the no-Tdap group, that could lead to conservative estimates of the association between vaccination and incident ADRD. We lacked biomarker and pathology data to confirm whether patients developed AD or other forms of dementia or both. However, clinician assessment has been shown to be accurate in identifying dementia cases in Down Syndrome [37]. As the use of biomarkers and neuroimaging become more common in diagnosing dementia, the disease may be diagnosed earlier and more accurately in the Down Syndrome population [38]. Given the sophistication of prenatal screening and postnatal identification of genetic anomalies, misclassification of Down Syndrome diagnoses is unlikely. We do not have measures about beliefs or attitudes toward vaccination and there is a possibility that healthy patient bias impacted results. This means persons who use more health care and obtain preventive medical care are likely less likely to develop dementia but more likely to receive vaccination. To address this concern we balanced health services use between vaccinated and non-vaccinated patients which reduces concern that more contact with healthcare confoundes the relationship between Tdap vaccination and risk for ADRD. We measured receipt of Tdap and not tetanus vaccination alone. This could have led to misclassifying exposure, yet post-hoc analyses suggests otherwise. We observed that only 26 patients (0.4%) of the sample had a tetanus vaccination. Among those without Tdap the prevalence was 0.1% and among those with Tdap the prevalence of tetanus only was 4.4%. It is unlikely that this biased results because only 0.1% of the non-Tdap group was exposed to tetanus vaccination. It is possible that we misclassified those who did not receive Tdap if they obtained it in a health care system not part of the TrinetX data base. If we classified vaccinated persons as unvaccinated, this could lead to underestimating the strength of association between Tdap and ADRD. Unmeasured confounding could bias results. For instance, we lack data on social support and access to care which are related to seeking preventive healthcare. Yet, the e-value was 2.04 which means an unmeasured confounder would require an association of this magnitude with vaccination status and incident ADRD to account for all results. It is unlikely such a confounder exists, as Appendix e-Table 3 shows that there is no single confounder that has a relationship of OR>2.0 (or <0.5) with both Tdap vaccination and HR>2.0 (or <0.5) for time to dementia. In addition, null results from negative control analyses further limit concerns about unmeasuerd confounding. We did not have mortality data to compute competing risk models and bias related to mortality could be present if there was a systematic difference in lifespan between vaccination groups. Thus we are not able to conclude that Tdap vaccination has a causal, preventive effect on incident ADRD. Last, the study was conducted in the United States and findings may not apply to other regions of the world.

## 6 Conclusions

This study identifies Tdap vaccination as an effective means of lowering the risk of incident ADRD in people with Down Syndrome. Health care providers should be vigilant in assuring that adults with Down Syndrome are given all age-appropriate vaccinations. They should also work with families to overcome any vaccine hesitancy. Emphasizing the second benefit of Tdap – lowering the risk of ADRD – may improve the rates of uptake. Further research should be undertaken to determine if other vaccines such as seasonal influenza or herpes zoster also have a protective effect against ADRD, and whether any effect is additive to the protection afforded by Tdap. If replicated, mechanisms underlying this relationship need to be identified.

## Sources of funding

Family and Community Medicine Department funds.

## Consent statement

A waiver of consent was granted because all data are historical and de-identified. There is no active participation by patients in this retrospective cohort study.

## Disclosures

None related to the topic of this manuscript.

## Declaration of generative AI and AI-assisted technologies in the writing process

AI was not used in any form or for any reason.

## Data statement

The authors do not have rights to share the data. People interested in accessing the cohort should contact TriNetX.

## CRediT authorship contribution statement

**Kimberly Schiel:** Writing – review & editing, Writing – original draft, Resources, Project administration, Methodology, Investigation, Funding acquisition, Conceptualization. **Joanne Salas:** Writing – review & editing, Writing – original draft, Resources, Methodology, Investigation, Formal analysis, Data curation, Conceptualization. **Anjani Urban:** Writing – review & editing, Writing – original draft, Investigation, Conceptualization. **Daniel F. Hoft:** Writing – review & editing, Writing – original draft, Methodology, Investigation, Conceptualization. **Jeffrey F. Scherrer:** Writing – review & editing, Writing – original draft, Supervision, Resources, Project administration, Methodology, Investigation, Conceptualization.

## Declaration of competing interest

The authors declare that they have no known competing financial interests or personal relationships that could have appeared to influence the work reported in this paper.

## Appendix Supplementary materials

Image, application 1
