# Supplementary material for: Tetanus, diphtheria and pertussis vaccination and risk for incident dementia among adults with down syndrome
Source: J Prev Alzheimers Dis. 2026 May 5;13(7):100583. doi: 10.1016/j.tjpad.2026.100583 (PMC13158761; doi:10.1016/j.tjpad.2026.100583)
Supplement: Supplementary file 1 [file mmc1.docx]

**APPENDIX**

| **e-Table 1. Variable definitions** | |
| --- | --- |
| **Variable** | **Definition** |
| Index/baseline | -6 possible index dates: 1/1/15, 1/1/16…..1/1/20  - First date where a patient is: 1) at least 40 years old; 2) without an indication of dementia; 3) regular healthcare user in 2-years prior (yearly ambulatory visit) |
| Follow-up time | Months from index to study end date (incident dementia or censoring). Censoring is date of last encounter available for those without incident dementia. |
| Tdap vaccination | Measured anytime prior to index date.   1. CPT: 90701, 90715 2. Drug name: Boostrix, Adacel |
| **Outcome and Exclusions** | |
| Dementia Exclusion Codes (used in washout) | \| **ICD-9 – Codes** \| \| \| \| \| \| \| \| \| --- \| --- \| --- \| --- \| --- \| --- \| --- \| --- \| \| 046.1x \| 046.3 \| \| \| 290.x \| \| 291.1 \| 291.2 \| \| 294.0 \| 294.1x \| \| \| 294.2x \| \| 331.x \| 332.x \| \| 333.0 \| 333.4 \| \| \| 438.0 \| \| 780.93 \| 797 \| \| **ICD-10 – Codes** \| \| \| \| \| \| \| \| \| A81.0x \| \| A81.2 \| F01.x \| \| F02.x \| \| F03.x \| \| F04 \| \| F05 \| F10.26 \| \| F10.27 \| \| F10.96 \| \| F10.97 \| \| G10 \| G20 \| \| G21.1x \| \| G21.2 \| \| G21.3 \| \| G21.4 \| G21.8 \| \| G21.9 \| \| G23.x \| \| G30.x \| \| G31.0x \| G31.1 \| \| G31.2 \| \| G31.83 \| \| G31.84 \| \| G31.85 \| G31.89 \| \| G31.9 \| \| G91.x \| \| G93.7 \| \| G94 \| I69.91 \| \| R41.2 \| \| R41.3 \| \| R41.81 \| \|  \|  \| \|  \| \|  \| |
| Dementia Main Outcome Codes | \| **ICD-9 – Codes** \| \| \| \| \| \| \| \| \| \| \| \| \| --- \| --- \| --- \| --- \| --- \| --- \| --- \| --- \| --- \| --- \| --- \| --- \| \| 290.0 \| \| 290.1x \| \| 290.2x \| \| 290.3 \| \| 290.4x \| \| \| 294.1x \| \| 294.2x \| \| 331.0 \| \| 331.1x \| \| 331.2 \| \|  \| \| \|  \| \| **ICD-10 – Codes** \| \| \| \| \| \| \| \| \| \| \| F01.x \| F02.x \| \| F03.x \| \| G30.x \| \| G31.0x \| \| G31.1 \|   - 2 code occurrences (on different days) within the same 12 month period  - Date of dementia onset is the first date of diagnosis when criteria met |
| Dementia medications | Donepezil, Rivastigmine, Galantamine, Memantine |
| **Comorbidity covariates – measured in the 2-years prior to index. At least one diagnostic code occurrence, unless otherwise stated. ‘X’ symbolizes a wildcard** | |
| Type II Diabetes | ICD-9 code: 250.x0, 250.x2, 357.2, 362.0x, 366.41;  ICD-10 code: E11.x, E08.42, E09.42, E13.42, E08.36, E09.36, E13.36  - 2 code occurrences in 24-months |
| Obesity | ICD-9 code: 278.00, 278.01; 278.03; V85.3; V85.4;  ICD-10 code: E66.01, E66.1, E66.2, E66.8, E66.9, Z68.3, Z8.4;  BMI ≥ 30 |
| Hypertension | ICD-9 code: 401.x;  ICD-10 code: I10 |
| Stroke or cerebrovascular accident | ICD-9 code: 431.x, 434.x, 438.x;  ICD-10 code: I61.x, I63.3x, I63.4x, I63.5x, I63.6, I63.8, I63.9, I66.x, I69.1x, I69.2x, I69.3x, I69.8x, I69.9x |
| Ischemic heart disease | ICD-9 code: 410.x – 414.x;  ICD-10 code: I20.x – I25.x |
| Congestive heart failure | ICD-9 code: 398.91, 402.11, 402.91, 404.11, 404.13, 404.91, 404.93, 428.x;  ICD-10 code: I09.81, I11.0, I13.0, I13.2, I50.x |
| Atrial fibrillation of flutter | ICD-9 code: 427.3x;  ICD-10 code: I48.x |
| Congenital heart defect | ICD-9 code: 745.x -747.x  ICD-10 code: Q20.x – Q26.x |
| Hypothyroidism | ICD-9 code: 243, 244.2, 244.3, 244.8, 244.9;  ICD-10 code: E02, E03.x |
| Traumatic brain injury | ICD-9 code: 800.x-804.x, 850.x-854.x, 905.0, , 907.0, 959.01, V15.52;  ICD-10 code: S01.90x, S02.x, S04.x, S06.x, S07.x, S09.8x, S09.9x, Z87.820 |
| Vitamin B12 deficiency | ICD-9 code: 266.2, 281.1  ICD-10 code: E53.8, D51.x |
| Depression | ICD-9 code: 296.2x, 296.3x, 311;  ICD-10 code: F32.0-F32.5, F32.9, F33.0-F33.3, F33.4x, F33.9  - 2 outpatient occurrences (on different days) in same 12-month period or 1 inpatient occurrence |
| Any anxiety disorder | ICD-9 code: 309.81, 300.00, 300.09, 300.01, 300.02, 300.23, 300.3;  ICD-10 code: F40.1x, F41.0, F41.1, F41.8, F41.9, F42, F43.1x  - Composite of PTSD, panic disorder, anxiety disorder not otherwise specified, obsessive compulsive disorder, social phobia, and generalized anxiety disorder  - 2 outpatient occurrences (on different days) in same 12 month period or 1 inpatient occurrence on/prior to index date. |
| Severe mental illness | ICD-9 code: 296.0x, 296.1x, 296.4x-296.8x, 298.9x, 295.x;  ICD-10 code: F30.x, F31.x, F20.x, F25.x  - Composite of bipolar disorder or schizophrenia |
| **Demographics and healthcare utilization.** | |
| High health services utilization | - Controls for detection bias related to more healthcare encounters.  - Average number of outpatient clinic visits per month, calculated as total visits divided by 24 months (i.e., the 2-year measurement period). The distribution of average number of outpatient clinic visits per month is dichotomized into high utilizer, >75^th^ percentile vs low utilizer, ≤75^th^ percentile. |
| Age | Age at index. The range in current data is 40-84. |
| Race | White, Black, Other, unknown |
| Sex | Male, female, unknown |
| Region | Variable provided in dataset based on census region. Categorized as Midwest, northeast, south, west, unknown |

| **e-Table 2. Demographic and baseline characteristics by Tdap vaccination status at index, after applying entropy balancing weights (n=5,591)** | | | | |
| --- | --- | --- | --- | --- |
| **Covariates** | **No Tdap**  **(n=5063)** | **Tdap**  **(n=528)** | **p-value** | **SMD%** |
| Age, mean (±sd) | 50.0 (±8.3) | 50.0 (±8.3) | .999 | 0.01 |
| Sex |  |  |  |  |
| Female | 50.1 | 50.1 | 1.0 | 0.00 |
| Male | 44.3 | 44.3 |  | 0.01 |
| Unknown | 5.6 | 5.6 |  | 0.02 |
| Race |  |  | 1.0 |  |
| White | 72.1 | 72.1 |  | 0.02 |
| Black | 10.1 | 10.1 |  | 0.02 |
| Other | 7.5 | 7.5 |  | 0.00 |
| Unknown | 10.3 | 10.3 |  | 0.01 |
| Region |  |  |  |  |
| Midwest | 19.1 | 19.1 | 1.0 | 0.01 |
| Northeast | 39.7 | 39.7 |  | 0.01 |
| South | 24.9 | 24.9 |  | 0.02 |
| West | 11.7 | 11.7 |  | 0.01 |
| Unknown | 4.6 | 4.6 |  | 0.02 |
| High healthcare utilization | 25.3 | 25.3 | .999 | 0.00 |
| Type II Diabetes | 10.0 | 10.0 | .999 | 0.01 |
| Obesity | 33.3 | 33.3 | .998 | 0.01 |
| Hypertension | 12.4 | 12.4 | .997 | 0.02 |
| Stroke | 1.6 | 1.6 | .998 | 0.01 |
| Ischemic heart disease | 2.7 | 2.7 | .998 | 0.01 |
| Congestive heart failure | 4.1 | 4.1 | .998 | 0.01 |
| Atrial fibrillation | 1.8 | 1.8 | .996 | 0.02 |
| Congenital heart defect | 8.4 | 8.4 | .999 | 0.00 |
| Hypothyroidism | 29.1 | 29.0 | .998 | 0.01 |
| Traumatic brain injury | 1.4 | 1.4 | .998 | 0.01 |
| Vitamin B12 deficiency | 2.3 | 2.3 | .994 | 0.03 |
| Depression | 7.3 | 7.3 | .996 | 0.02 |
| Anxiety disorder ^a^ | 9.6 | 9.6 | .999 | 0.00 |
| Severe mental illness ^b^ | 2.2 | 2.2 | .997 | 0.02 |
| NSAIDs^c^ | 14.6 | 14.6 | .997 | 0.02 |
| Antihypertensives^c^ | 14.7 | 14.7 | .999 | 0.00 |
| Statins^c^ | 9.6 | 9.6 | .999 | 0.01 |
| Steroids^c^ | 18.3 | 18.3 | .999 | 0.01 |
| Antivirals^c^ | 3.2 | 3.2 | .998 | 0.01 |
| Metformin^c^ | 3.2 | 3.2 | .999 | 0.00 |
| Sulfonylurea^c^ | 1.2 | 1.2 | .993 | 0.04 |
| Antidepressant medications^c^ | 11.7 | 11.7 | .998 | 0.01 |
| Atypical antipsychotics^c^ | 5.1 | 5.1 | .999 | 0.00 |
| Benzodiazepines^c^ | 8.7 | 8.8 | .996 | 0.02 |
| Note: SMD%=standardized mean difference percent (SMD*100)  ^a^Anxiety disorders = panic disorder, OCD, social phobia, GAD, Anxiety NOS, PTSD  ^b^ Severe mental illness = bipolar disorder or schizophrenia  ^b^Medications = sustained use prior to index (at least 2 prescription orders in any 6-month period) | | | | |

| **Appendix e-Table 3. Crude relationship of each covariate with vaccine status and time to dementia (n=5,591)** | | |
| --- | --- | --- |
| **Covariates** | **Tdap (y/n)**  **OR (95% CI)^a^** | **Time to dementia**  **HR (95% CI)^b^** |
| Age | 0.95 (0.94-0.96) | 1.05 (1.04-1.06) |
| Sex |  |  |
| Female | 1.00 | 1.00 |
| Male | 0.83 (0.69-0.99) | 2.41 (1.88-3.10) |
| Race |  |  |
| White | 1.00 | 1.00 |
| Black | 1.12 (0.84-1.50) | 0.85 (0.63-1.14) |
| Other | 1.10 (0.79-1.53) | 1.20 (0.96-1.50) |
| Region |  |  |
| Midwest | 1.00 | 1.00 |
| Northeast | 0.47 (0.36-0.59) | 1.49 (1.22-1.82) |
| South | 0.68 (0.53-0.88) | 0.89 (0.70-1.14) |
| West | 0.90 (0.67-1.21) | 1.30 (1.00-1.70) |
| High healthcare utilization | 3.32 (2.76-3.98) | 1.09 (0.93-1.27) |
| Type II Diabetes | 1.20 (0.91-1.60) | 1.12 (0.89-1.40) |
| Obesity | 1.83 (1.53-2.19) | 0.80 (0.69-0.94) |
| Hypertension | 1.61 (1.27-2.04) | 0.72 (0.56-0.91) |
| Stroke | 0.71 (0.31-1.63) | 1.09 (0.61-1.92) |
| Ischemic heart disease | 1.76 (1.12-2.76) | 1.20 (0.79-1.84) |
| Congestive heart failure | 1.37 (0.91-2.06) | 0.92 (0.62-1.39) |
| Atrial fibrillation | 1.16 (0.62-2.19) | 0.51 (0.24-1.08) |
| Congenital heart defect | 1.25 (0.93-1.69) | 1.04 (0.81-1.34) |
| Hypothyroidism | 1.41 (1.17-1.70) | 1.66 (1.44-1.92) |
| Traumatic brain injury | 1.91 (1.05-3.49) | 0.63 (0.28-1.42) |
| Vitamin B12 deficiency | 2.65 (1.72-4.09) | 1.20 (0.78-1.86) |
| Depression | 2.87 (2.22-3.71) | 0.85 (0.64-1.13) |
| Anxiety disorder | 2.55 (2.02-3.24) | 0.83 (0.65-1.08) |
| Severe mental illness | 1.45 (0.85-2.47) | 0.84 (0.49-1.42) |
| NSAIDs | 2.01 (1.62-2.50) | 0.60 (0.47-0.76) |
| Antihypertensives | 1.25 (0.99-1.59) | 0.68 (0.54-0.86) |
| Statins | 1.08 (0.81-1.46) | 1.22 (0.98-1.53) |
| Steroids | 1.34 (1.08-1.66) | 0.87 (0.72-1.06) |
| Antivirals | 2.39 (1.62-3.51) | 0.55 (0.33-0.94) |
| Metformin | 1.30 (0.81-2.06) | 0.83 (0.54-1.27) |
| Sulfonylurea | 1.70 (0.86-3.34) | 1.23 (0.66-2.29) |
| Antidepressant medications | 1.77 (1.39-2.25) | 1.08 (0.87-1.33) |
| Atypical antipsychotics | 1.10 (0.74-1.63) | 1.36 (1.01-1.82) |
| Benzodiazepines | 1.05 (0.77-1.43) | 0.88 (0.67-1.16) |
| ^a^ Logistic regression model  ^b^ Cox proportional hazard model | | |
